# Glaucony authigenesis, maturity and alteration in the Weddell Sea: An indicator of paleoenvironmental conditions before the onset of Antarctic glaciation

**DOI:** 10.1038/s41598-019-50107-1

**Published:** 2019-09-19

**Authors:** Adrián López-Quirós, Carlota Escutia, Antonio Sánchez-Navas, Fernando Nieto, Antonio Garcia-Casco, Agustín Martín-Algarra, Dimitris Evangelinos, Ariadna Salabarnada

**Affiliations:** 1 Instituto Andaluz de Ciencias de la Tierra, CSIC-Universidad de Granada, Avda. las Palmeras 4, 18100 Armilla, Granada Spain; 2 Department of Mineralogy and Petrology, University of Granada, 18071 Granada, Spain; 3 Department of Stratigraphy and Paleontology, University of Granada, 18071 Granada, Spain

**Keywords:** Palaeoclimate, Marine chemistry

## Abstract

Three types of glaucony grains were identified in the late Eocene (~35.5–34.1 Ma) sediments from Ocean Drilling Program (ODP) Hole 696B in the northwestern Weddell Sea (Antarctica). The grains are K_2_O-rich (~7 wt%) and formed by smectite-poor interstratified ~10 Å glauconite-smectite with flaky/rosette-shaped surface nanostructures. Two glaucony types reflect an evolved (types 1 and 2 glaucony; less mature to mature) stage and long term glauconitization, attesting to the glaucony grains being formed *in situ*, whereas the third type (type 3 glaucony) shows evidences of alteration and reworking from nearby areas. Conditions for the glaucony authigenesis occurred in an open-shelf environment deeper than 50 m, under sub-oxic conditions near the sediment-water interface. These environmental conditions were triggered by low sedimentation rates and recurrent winnowing action by bottom-currents, leading to stratigraphic condensation. The condensed glaucony-bearing section provides an overview of continuous sea-level rise conditions pre-dating the onset of Antarctic glaciation during the Eocene-Oligocene transition. Sediment burial, drop of O_2_ levels, and ongoing reducing (postoxic to sulphidic) conditions at Hole 696B, resulting in iron-sulphide precipitation, were a key limiting factor for the glauconitization by sequestration of Fe^2+^.

## Introduction

Glauconite is the iron-potassium hydrous phyllosilicate mineral typical of the glaucony green marine clay facies, which also includes Fe-rich, 2:1 dioctahedral specimens with expandable layers of randomly interstratified glauconite-smectite^1^. The term glaucony was defined first by Odin and Létolle^2^ for marine green sediments formed by mixed layer glauconite-smectite minerals, which differ from the nonexpanding end-member glauconitic mica (i.e. the true glauconite mineral). Glaucony is commonly reported in low-latitude, shallow-marine settings at water depths <500 m, temperatures below 15 °C, and under sub-oxic, partially reducing conditions^3^. It is typically associated with low sedimentation rates and condensed intervals^3^. However, glaucony has also been reported in deep-sea (>2000 m), low-temperature (3–6 °C) environments^4–6^, and in very shallow marine, estuarine^7–9^ and paleosol^9^ settings. Most of the aforementioned settings share several post-depositional physicochemical conditions: (1) low accumulation rates of detrital sediment, (2) long residence times (10^3^–10^6^ years for ancient records^3^, and alternatively <10^4^ years for recent records^10^) of the detrital grains near to the sediment-water interface, (3) granular substratum (siliciclastic or carbonatic; including the muddy filling inside the cavities of carbonate bioclasts) with high permeability and porosity, (4) redox potential (Eh) ~0 mV, (5) seawater pH 7–8, and (6) organic matter-rich, semiconfined micro-environments. In summary, glaucony is a sensitive proxy of low sedimentation rates in the marine realm and constitutes a powerful tool for sedimentological and sequence stratigraphic interpretations, due to its association with well-defined trends of sea-level change^11,12^.

Glaucony authigenesis in Antarctica has received little attention to date when compared with other mid- to low-latitude Cenozoic to present glaucony records^13^. Cenozoic glaucony-bearing facies have been widely reported in Antarctica and the Southern Ocean (e.g. Tasman Region^14^; Falkland Plateau^15^; Seymour Island^16^; South Orkney Microcontinent (SOM)^17^; Great Australian Bight^18^; Otway Basin^19^; Supplementary Fig. S1). In spite of all these reported occurrences, the genesis, depositional setting control and paleoenvironmental implications of glaucony in these Antarctic regions are loosely constrained. This information, is however relevant because the occurrence of glaucony around the Antarctic margin has been noted in late Eocene sedimentary sequences^20^. Antarctic glaucony deposits thus provide an opportunity to study the poorly-understood paleoenvironmental conditions prior to one of the major climatic transitions in the Cenozoic, the Eocene-Oligocene transition (EOT, ~34–33.6 Ma). The EOT is marked by a rapid increase in benthic foraminifer δ^18^O values^21^ involving cooling and the growth of a continent-wide Antarctic ice sheet by the earliest Oligocene (oxygen isotope event Oi-1, ~33.6 Ma)^22^.

**Figure 1:**
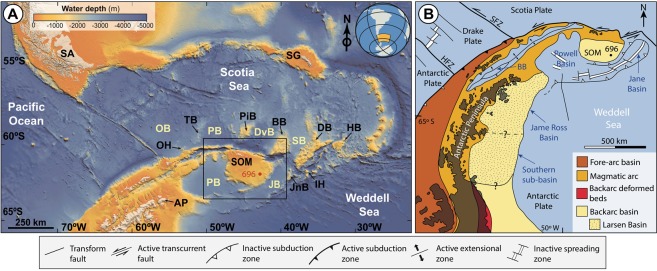
Geological setting of the Drake-Passage Scotia Sea region. (**A**) Simplified bathymetry map derived from GEBCO, showing the small basins connecting the Weddell Sea with the Scotia Sea. The squared area indicates the study area (see detailed map in Supplementary Fig. S1A). *OB: Ona Basin; OH: Ona High; TB: Terror Bank; PB: Protector Basin; PiB: Pirie Bank; DvB: Dove Basin; BB: Bruce Bank; SB: Scan Basin; DB: Dove Bank; PB: Powell Basin; JB: Jane Basin; JnB: Jane Bank*. (**B**) Geotectonic sketch map with lithotectonic units superimposed for the northern Antarctic Peninsula and the South Orkney Microcontinent region. Lithotectonic units displayed in the sketch map are from Elliot^63^.

Previous work suggests that the initial deepening of the seaway at the EOT led to the isolation of the SOM from the Antarctic Peninsula^17^. Based on geophysical studies of the region, the western and southern-to-southeastern margins of the SOM result from continental rifting and subsequent opening of Powell and Jane basins during the Cenozoic^23^ (Fig. 1). Jane basin is a backarc basin related to subduction of the Weddell oceanic lithosphere below the south and southeastern margins of the SOM^24^. Furthermore, hydrothermal circulation associated with the Powell Ridge spreading axis has been reported^25,26^. Whilst the geodynamic setting of the SOM is fairly well constrained^23^ (for details see Supplementary Information), the timing of events and the paleoenvironmental conditions throughout the Cenozoic remain obscure.

In this contribution we describe, for the first time in the Southern Ocean, a significant Cenozoic (late Eocene) glauconitization event in the vicinity of the SOM shelf (Site 696^17^; Fig. 1 and Supplementary Fig. S2A), utilizing textural, mineralogical and geochemical analyses. Based on this multi-proxy approach, we reconstruct the paleoenvironmental conditions, including water depth changes, sediment-water interface (before burial) oxygenation conditions, and the influence of post-depositional alterations on authigenic mature glaucony. Altogether, this information provides an important window into the paleoenvironmental conditions prevailing in this Antarctic Peninsula-SOM region before major ice sheet advance during the EOT.

## Material and Methods

The ODP Leg 113, Hole 696B (latitude: 61°50.959′S, longitude: 42°55.996′W) was drilled on the SOM in the northwestern Weddell Sea, at 650 m water depth (Fig. 1 and Supplementary Fig. S2A). The recovered sedimentary column (645.6–0 mbsf) consists of three main sediment types (terrigenous, diatomaceous and hemipelagic) spanning the late Eocene to the Quaternary^17,27^ (see Supplementary Information; Supplementary Fig. S2B). Based on dinocyst stratigraphy, Houben *et al*.^28^ determined the depth of sediments recording the EOT and the Oi-1 event from 571.5 mbsf to 569.1 mbsf.

This study focuses on sediments containing high amounts of glaucony grains recovered between 606.9–569.7 mbsf (*Subunit VIIC* in Barker *et al*.^17^; Supplementary Fig. S2B) therefore predating the EOT (late Eocene ~35.5–34.1 Ma), based on the existing age model (Supplementary Information and Supplementary Fig. S2B). Glaucony-bearing samples were selected from different stratigraphic positions throughout the poorly investigated glauconitic packstone facies (Cores 57R-56R; Supplementary Fig. S2C), in order to study potential textural and compositional differences between the glaucony grains. Six thin sections, about 30 µm thick, were prepared by epoxy impregnation in order to fill the pores and consolidate the poorly lithified glauconitic packstone samples selected from cores (Supplementary Fig. S2C). In addition, glaucony grains were extracted from the whole rock core samples after sieving fractions of 125–250 µm and 250–500 µm. The coarse sediment fractions were later separated by universal electromagnetic separator UMC-1. Purified glaucony grains were further separated from diagenetic complex growths and aggregates along with other sediment grains under the binocular microscope.

**Figure 2:**
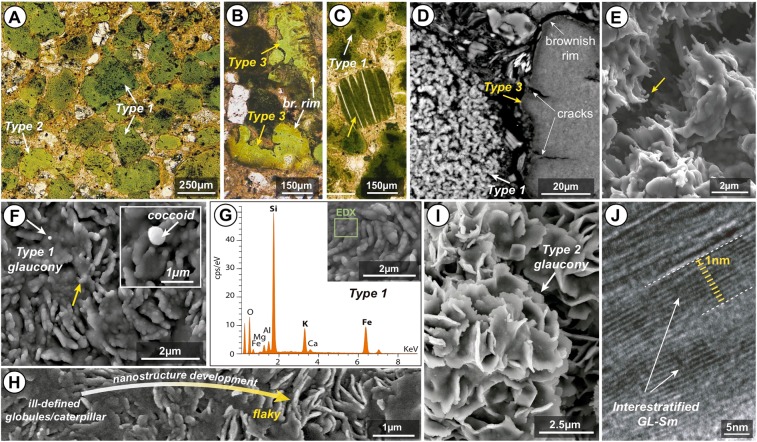
Glauconitic packstone facies. (**A,B)** Plain-polarized light (PPL) photomicrographs of glaucony grains of types 1, 2 and 3. (**C**) PPL photomicrograph of a glauconitized mineral (mica)-grain (yellow arrow). (**D**) SEM photomicrograph (BSE) showing the spotty flaky texture in section of a type 1 glaucony grain (white arrow) and the smooth interior of type 3 glaucony (yellow arrow). Note cracks and poorly altered rims associated with type 3 glaucony. (**E**,**F**) SEM photomicrographs (SE) showing the nanostructure of the surface of type 1 glaucony, with probable bacterial threads (ill-defined globules/caterpillar structures; yellow arrow) and coccoid-like forms (white arrows). (**G**) Energy-dispersive X-ray (EDX) analysis of type 1 glaucony. (**H**) SEM photomicrographs (SE) showing the development of type 1 glaucony nanostructure from ill-defined globules/caterpillar to low packing size/density lamellar-flaky. (**I**) SEM photomicrograph (SE) showing type 2 glaucony nanostructure with well developed evolved flaky honeycombed-shaped structures. (**J)** HRTEM image showing well-defined ~10Å lattice fringes of glauconite crystal and related interstratified smectitic area.

Isolated glaucony grains were studied first under binocular microscope for morphological characterization (see Supplementary Fig. S4B,C). In addition, the textures of the glaucony-bearing packstone facies and of the isolated glaucony grains were examined under petrographic and scanning- and transmission-electron microscope (Fig. 2; Supplementary Figs S3D, S4, S5, S6A). During the petrographic study, several photomicrographs of the glaucony-bearing facies were taken with an OLYMPUS DP20 camera connected to a petrographic OLYMPUS BX60 microscope and captured with the OLYMPUS DP image-management software (Instituto Andaluz de Ciencias de la Tierra, CSIC-UGR). Back-scattered electron (BSE) and secondary electron (SE) images of the glaucony-bearing facies and isolated glaucony grains were obtained with an environmental scanning electron microscope (ESEM) FEI Quanta 400 (CIC, University of Granada). High-resolution transmission electron microscopy (HRTEM) photomicrographs were obtained with a Titan instrument with XFEG emission gun, spherical aberration corrector and HAADF detector, working at 300 kV, with a resolution of 0.8 Å in the HRTEM mode and 2 Å in the scanning transmission electron microscopy (STEM) mode (CIC, University of Granada). Selected-area electron diffraction (SAED) patterns were acquired for glaucony and other authigenic mineral packets with the same instrument.

X-ray-diffraction (XRD) diagrams from powder glaucony grain concentrates (reduced in size with an agate mortar) and oriented aggregates of the <2 µm fraction treated with ethylene glycol, were recorded using a PANalytical X’Pert Pro diffractometer (CuKα radiation, 45 kV, 40 mA) equipped with an X’Celerator solid-state linear detector (University of Granada). Data were collected for 10 sec in 0.008° 2θ steps. In the case of superposed peaks, the identification of phases and the measurement of intensities of each individual peak were carried out with the help of decomposition routines included in the HighScore software. Qualitative elemental analyses were also obtained through the aforementioned ESEM FEI Quanta 400 (CIC, University of Granada), equipped with an energy-dispersive X-ray spectroscopy system (EDX).

Electron probe microanalysis (EPMA) of the main glaucony-forming elements was carried out with a CAMECA SX100 automated electron microprobe (CIC, University of Granada) in the wavelength dispersive mode under the following conditions: acceleration voltage 15 kV, probe current 15 nA, and an electron beam diameter 5 µm allowing point-by-point element determination. In addition, elemental XR images were obtained with the same CAMECA SX100 instrument operated at 20 kV and 205 nA beam current, with step (pixel) size of 2 μm and counting time of 45 ms. The images were processed with Imager software (R. L. Torres-Roldán & A. García-Casco, unpublished) and consist of the XR signals of Kα lines of the elements (colour-coded; expressed in counts) and with voids, polish defects, and all other mineral phases masked out, overlain onto a grey-scale base-layer calculated with the expression ∑ [(counts/nA per s)i·Ai], (where A is atomic number, and i is Si, Ti, Al, Fe, Mn, Mg, Ca, Ba, Na, K, P, S and O), which contains the basic textural information of the scanned areas.

The same powder used for the XRD analyses was also used for the spectrometric study of color. The coexistence of both Fe^2+^ and Fe^3+^ in the structure of silicate minerals is responsible for the occurrence of light absorption that affects their colour^29^. Diffuse reflectance spectra in the ultraviolet – visible – near infrared (UV–VIS–NIR) were recorded from powder samples at room temperature on a Varian Cary 5E UV–VIS–NIR spectrophotometer (200–2000 nm) (CIC, University of Granada).

## Results

Green-clay authigenesis within the late Eocene glauconitic packstone facies at ODP Hole 696B (Supplementary Figs S2B–C, S3A–E, S4A), has occurred mainly by the transformation of pellets (glauconitized grains of 125–500 μm; Fig. 2A,B; Supplementary Figs S4, S5) set in sandy to silty smectite-rich, mixed terrigenous-carbonatic sediments (Supplementary Fig. S3A–E). Besides siliceous biogenous and authigenic components, the major mineral components found into clay size fractions are quartz, alkali feldspar and clay minerals (Supplementary Fig. S3B). Previous studies^17,30^ identified and quantified these clay minerals as smectite (common to exclusive: >70%) and illite (rare to abundant: <20%), associated with sporadic chlorite and kaolinite (rare to common: ~10%). In addition, we observe very rare dark green, oriented microcrystalline glauconitized mineral (mica)-grains with high birefringence and parallel cleavage (accordion-like green grains: Fig. 2C; Supplementary Fig. S4B). Three population types of glaucony grains have been distinguished in the sediments under study: *Type 1 glaucony* is formed by rounded olive green grains without cracks (Fig. 2A,D; Supplementary Fig. S4B,C), with rough surfaces with low packing size/density lamellar-flaky nanostructure. Type 1 glaucony often preserve ill-defined globules and caterpillar nanostructures^31^. These nanostructures most resemble bacterial threads and coccoid-like forms (Fig. 2E–H; Supplementary Fig. S4D) similar to those described in other studies^11,32^, but are also comparable with those of silica microtexture surfaces^33^. These grains show internal spotty to flaky texture in section, as shown by BSE images (Fig. 2D; Supplementary Fig. S5E) and by plain-polarized light images (Supplementary Fig. S5A–C). *Type 2 glaucony* is made of rounded mammillated to lobate (cerebroid), dark green grains with smooth surfaces with flaky honeycombed nanostructure (Fig. 2A,I; Supplementary Fig. S4F) and often cracked at the margins (Supplementary Figs S4C, S5C)*. Type 3 glaucony* shows brownish-greenish colours (Fig. 2B; Supplementary Fig. S5D) and sub-angular morphology, although cerebroid grains and grain fragments similar to type 2 glaucony are also common. These grains are frequently cracked, show smooth, uniform internal structure in BSE images and display gradual change from darker (brownish) to lighter (greenish) colour from rims to cores and, especially, along cracks (Fig. 2D).

**Figure 3:**
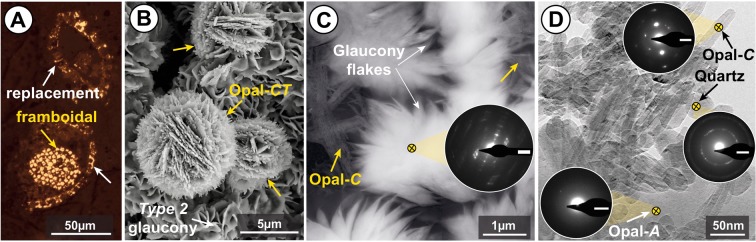
(**A**) Reflected light microscope (RLM) photomicrograph of pyrite framboids (yellow arrow) and pyritized formerly siliceous skeletons (white arrows). (**B**) SEM photomicrograph (SE) displaying silica lepispheres (opal-CT; yellow arrows) on the surface of type 2 glaucony. (**C**) High-resolution transmission electron microscope (HRTEM) photomicrograph of glaucony flakes (white arrows) and respective selected-area electron diffraction (SAED) pattern displaying intense [001] reflections centered near 10 Å, and of low cristobalite (opal-C; yellow arrow). (**D**) Silica mineral phases under HRTEM, with their respective SAED patterns: (1) opal-A, characterized by no diffracted intensities (i.e. amorphous silica); (2) opal-C, characterized by sharp, intense (101) reflections centered ~4.0 Å; and (3) microcrystalline quartz, depicted by (101) reflections centered near 3.3 Å. Scale bars (SAED patterns): 2 1/nm.

Other authigenic minerals present in the matrix between glaucony grains include the following, in addition to zeolite already described by Barker *et al*.^17^: (a) pyrite as framboidal aggregates and crystalline replacements of siliceous bioclasts (Fig. 3A; Supplementary Fig. S5G,H), and (b) silica minerals as amorphous silica (opal-A), lepispheres of opal-CT, low cristobalite (opal-C), and microcrystalline quartz (Fig. 3B–D). The occurrence of silica minerals is rarely observed in our SEM analysis, except for rare silica lepispheres found outside of glaucony grains (Fig. 3B). Silica particles were detected mainly by HRTEM analysis including electron diffraction (Fig. 3C,D).

**Figure 4:**
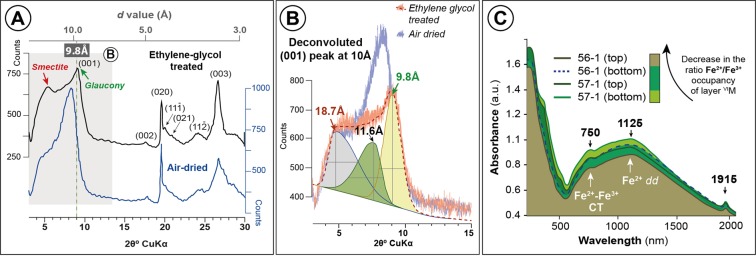
(**A**) Powder X-ray diagrams of air-dried and ethylene-glycol treated late Eocene glaucony concentrates, with indication of *d* value. Note strong reflection at ~9.8 Å of smectite-poor interstratified glauconite-smectite (green arrow), and the ~17 Å peak of a secondary smectite (red arrow). (**B**) Deconvoluted (001) peak at ~10 Å of ethylene-glycol-treated glaucony. Smectite-poor interstratified glauconite-smectite (green arrow), and secondary smectite (red arrow) are also indicated. Note that deconvoluted green and yellow peaks (reflections at 11.6 Å and 9.8 Å, respectively) correspond to R3 mica(0.9)/smectite (see for comparison Fig. 8.7 of Moore and Reynolds^33^). (**C**) UV–VIS–NIR spectra of glaucony grains from core samples 56R and 57R.

XRD air-dried spectrums of paramagnetic fraction rich in glaucony grains display a slightly higher-spacing reflection than expected for pure glauconite (~10.8 Å) according to the higher-order (*d*00l) basal peaks (e.g. (003); Fig. 4A). After ethylene-glycol treatment, this first basal reflection splits into various peaks, of which the highest intensity at ~9.8 Å spacing with a tale toward lower angles corresponds to a glauconite-smectite mixed layer^34^ (Fig. 4A,B). XRD data also indicates a significant secondary discrete smectite content, as evidenced by *d*001 peak splitting at ~17 Å after glicolation (Fig. 4A,B).

UV–Vis–NIR color spectroscopy of glaucony grains displays gradually decreasing levels of absorbance of both Fe^2+^–Fe^3+^ charge transfer and Fe^2+^ *dd* absorption bands (at 750 and 1125 nm respectively; Fig. 4C) in cores 57R to 56R. Furthermore, the absorption bands observed at 1915 nm, which denote presence of H_2_O molecules, also decrease upward through the glauconitic horizon (Fig. 4C). Color of Fe-bearing minerals is dependent on the occurrence of Fe^2+^/Fe^3+^ in their structures^35^. Thus, color and absorption spectroscopy reveals a gradually decreasing Fe^2+^:Fe^3+^ ratio in the occupancy of the octahedral layer of glaucony grains (Fig. 4C; see also Fig. 10 in Sánchez-Navas *et al*.^36^). This corresponds to a gradually decreasing abundance of types 1 and 2 glaucony along with a slight increase in brownish-green type 3 glaucony grains upwards through the studied core (Supplementary Fig. S2B).

**Figure 5:**
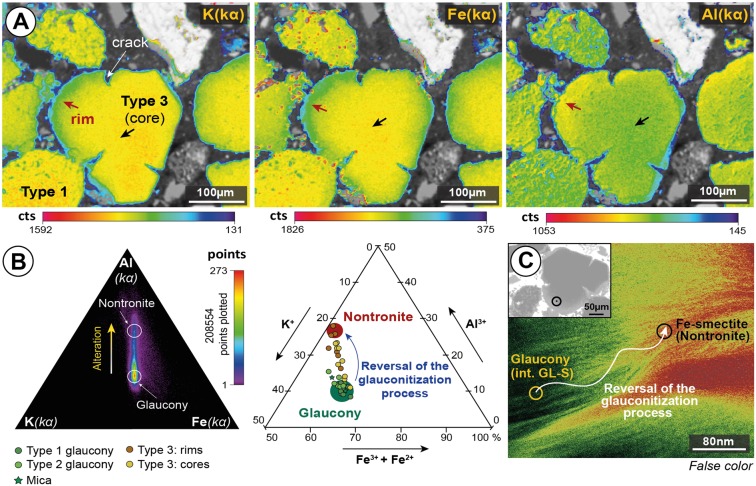
Chemical composition obtained by electron probe microanalysis (EPMA). (**A**) X-ray (Kα) mappings showing abundances of K, Fe and Al with voids, polish defects, and all other mineral phases masked out, overlain onto a grey-scale base-layer calculated with the expression ∑ [(counts)i·Ai], (where A is atomic number, and i is Si, Ti, Al, Fe, Mn, Mg, Ca, Ba, Na, K, P, S and O), which contains the basic textural information of the scanned areas. Color scales represent counts (cts) with warmer colors representing more intense X-ray signals. Altered rims (red arrows) have lower K and Fe contents, and higher Al content relative to cores (black arrows). (**B**) Triangular diagrams Al-K-Fe showing the chemical domains of glaucony and nontronite. First diagram to the left corresponds to the accumulated number of pixels obtained from X-ray (qualitative) maps presented in A. The second diagram to the right corresponds to single-spot quantitative EPMA analyses (a.p.f.u.). (**C**) HRTEM photomicrograph showing reversal of the glauconitization process. False color image (phase map) with colors corresponding to AEM compositions of: interstratified glauconite-smectite, (Si_3.53_Al_0.29_Fe^3+^_0.18_)_4_(Al_0_Mg_0.34_Fe_1.68_)_2.02_(K_0.77_Ca_0.03_)_0.8_ (green color), and nontronite, (Si_3.73_Al_0.27_)_4_(Al_0.30_Mg_0.41_Ti_0.03_Fe_1.30_)_2.02_ (K_0.43_Ca_0.03_)_0.46_ (red color).

**Figure 6:**
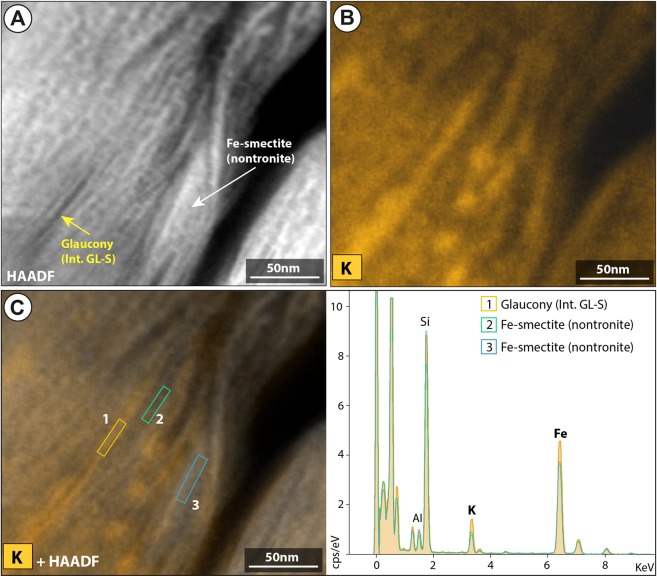
HRTEM EDX maps of potassium. (**A**) and (**B**) High-angle annular dark field (HAADF) STEM image and corresponding EDX map showing the distribution of K in the core-rim boundary of type 3 glaucony. (**C**) Combined EDX map of K and HAADF image with corresponding EDX spectra of the interstratified glauconite-smectite (core) and Fe(III)-smectite (nontronite rim). The yellow, green and blue boxes indicate the position of EDX analysis.

According to mineral chemistry analysis, type 2 displays slightly higher values in K_2_O, Fe_2_O_3_ (total Fe), and Al_2_O_3_ than type 1 (Table 1 and point-by-point analyses in Supplementary Table S1). Rims of type 3 grains show the lowest content of K_2_O and Fe_2_O_3_, and the highest in Al_2_O_3_ content compared to types 1 and 2 (Table 1 and point-by-point analyses in Supplementary Table S2). However, the values in the cores of type 3 grains range between those of the rims in the same grains and of the grains of types 1 and 2 values. The X-ray maps of K, Fe and Al were constructed to show the changes in compositions between green cores and brownish areas (in the rims and cracks) (Fig. 5A; Supplementary Fig. S6 and Table S2). Brownish rims/cracks of type 3 glaucony were revealed as a Fe-bearing dioctahedral smectite (nontronite) phase according to their textural, mineral and chemical characteristics. Furthermore, HRTEM images at the core-rim contact of type 3 glaucony display significant differences of composition (Fig. 6), which is compatible with the coexistence at the nanoscale of both glaucony and nontronite.

**Table 1:** Electron probe microanalysis (EPMA) of the glaucony types displaying the average K_2_O, Fe_2_O_3_* (total) and Al_2_O_3_ values (wt %).

Types (wt %)	1	2	3	
			*cores*	*rims*
K_2_O	6.96 (6.41–7.55)	7.28 (6.58–7.86)	5.80 (5.24–6.39)	5.07 (4.33–5.25)
Fe_2_O_3_*	27.02 (24.31–28.08)	27.45 (26.33–29.69)	23.62 (21.40–26.57)	20.56 (17.85–23.43)
Al_2_O_3_	2,91 (2.33–3.45)	3.39 (2.75–4.68)	5.23 (2.95–5.98)	5.80 (4.08–6.88)

*Total Fe expressed as Fe^3+^. Min-max values in parentheses. For complete information of the point-by-point EPMA microanalysis see Supplementary Tables S1 and S2.

## Discussion

### Paleoenvironmental implications of glaucony maturity

**Figure 7:**
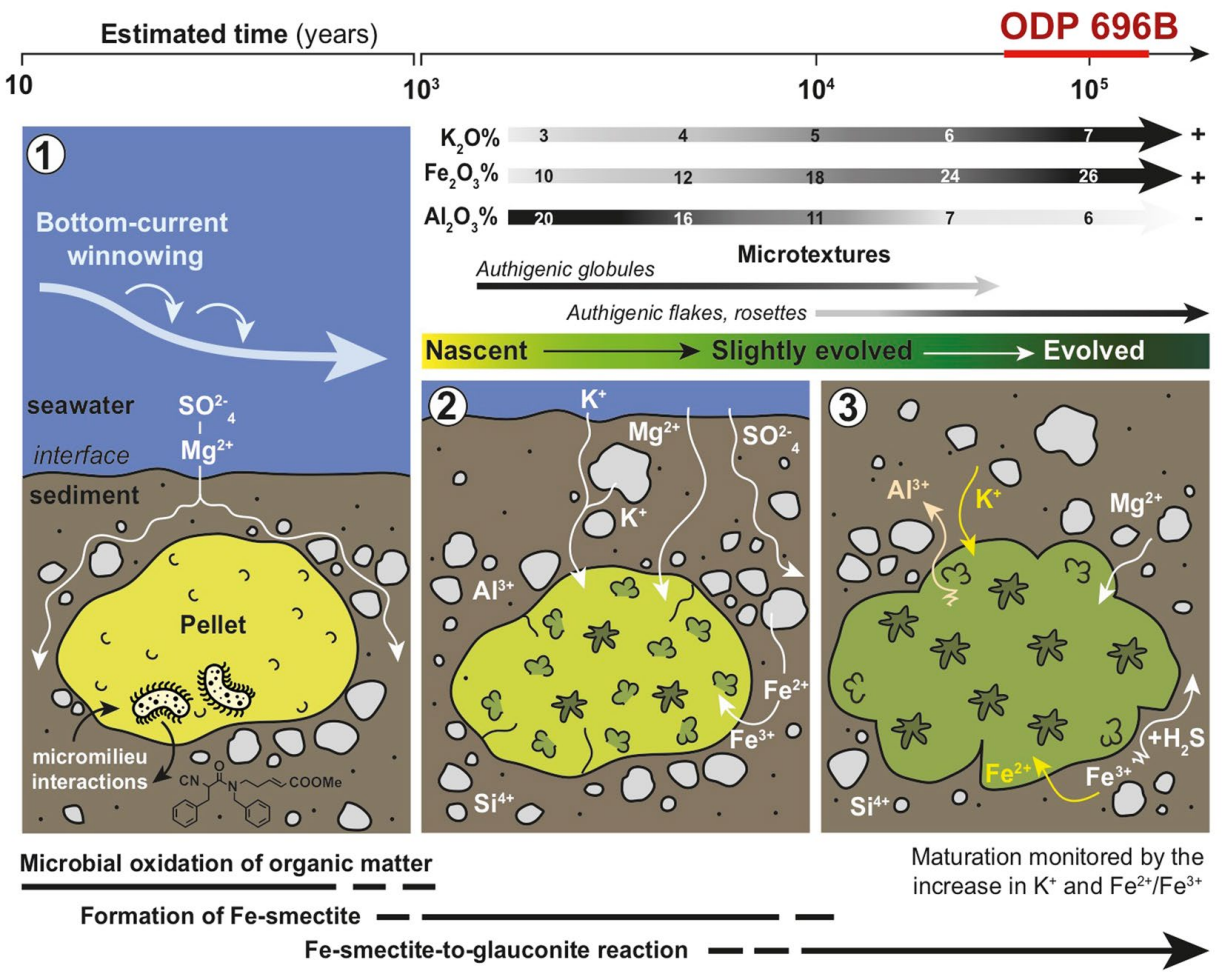
Model for glauconitization in the studied grains (following the ideas of Odin and Matter^31^; and Baldermann *et al*.^6^). **(1)** Microbial oxidation of organic matter (~10–10^3^ years). **(2)** Neoformation of Fe(III)-smectite precursor (~10^3^–10^4^ years). **(3)** Fe(III)-smectite to glauconite reaction (~10^4^–10^6^ years). Glaucony maturation involves the chemical change of Si, Al^VI^, Mg, Ca and Na by Al^IV^, Fe (Fe^2+^) and K, from smectitic glauconite (nascent stage) to glauconitic mica (highly evolved stage). Microtextures also attest glaucony maturation from globules and caterpillar structures, maybe related in origin to authigenesis of clays associated with bacterial structures (cf. Eder *et al*.^11^; Zanin *et al*.^32^; Sánchez-Navas *et al*.^36^) to the flaky honeycombed and rosette structures typical of green clay crystals.

The glauconitization process has been divided into four evolutionary stages according to specific morpho-textural, mineralogical and geochemical characteristics of glaucony grains^3,31,37^: (1) nascent, (2) slightly evolved, (3) evolved, and (4) highly evolved. Late Eocene ODP Hole 696B types 1 and 2 glaucony are interpreted to be mature (evolved: ~7% K_2_O; Table 1 and Fig. 7), smectite-poor interstratified ~10 Å glauconite-smectite (Figs 2 and 4A,B). The time required to produce an autochthonous evolved glaucony grain is considered to be higher than 10^5^ years for ancient glaucony-bearing records^3^. Whilst, Bornhold and Giresse^10^ estimated that recent glaucony off Vancouver Island, formed during the transgressive phase following the Last Glacial Maximum through the last 10^3^–10^4^ years. We interpret the moderately sorted, irregular-shaped types 1 and 2 glaucony grains at ODP Hole 696B to have been formed *in situ* (autochthonous *sensu* Amorosi^38^), in low-energy environments. The composition and surface texture of the type 1 grains, which show less crystalline surface textures and preserve bacterial-like morphologies similar to those described in comparable glauconitized settings^11,32^, indicate that they are less mature than type 2. Classic microfacies criteria^39^ allows to establish a relationship between the winnowing of fine-grained particles, sorting and rounding of grains and the intensity of hydrodynamic processes. According to their shape, sorting and proportion of grains and matrix (i.e. glauconitic packstone texture), types 1 and 2 glaucony grains may have been episodically winnowed and exposed at the sea bottom during a period of low sedimentation rate and stratigraphic condensation lasting for a few tens of thousands years (for details see Supplementary Information and Supplementary Fig. S7). When the glaucony is immature (nascent stage *sensu* Odin and Matter^31^), any transport experienced by the soft, clayey pellets can result in their disaggregation. Evolved, cracked glaucony grains would be also vulnerable to mechanical breakdown into smaller, less regular fragments during physical transport or intense bioturbation. Neither grain disaggregation nor mechanical breakdown is observed in the studied sediments. The type 2 cracked but non-fragmented glaucony therefore indicate that grains formed *in situ* and that low-energy conditions prevailed during glauconitization (Supplementary Figs S4C, S5C). This implies that glaucony at Hole 696B was preserved from burial for a long time, favouring increased maturity. The abundance of mature glaucony at Hole 696B is consistent with the formation of an autochthonous condensed section, as also described at several sites in Western Europe by Amorosi^38^.

The real nature of glauconitized substratums is often difficult to determine. Fecal pellets are the main substrate for glaucony formation in both recent and ancient sediments^13^. Recent fecal pellets are documented to be produced in shelf environments (<100 m water depth) by carbonate ingesting organisms including crustaceans and gastropods^39,40^, and contain vast quantities of undigested organic matter and clay- and silt-sized material^40^. Glauconitized pellets could be interpreted as fecal pellets in origin base on their specific morphology and internal texture^41^. Likewise, carbonate mud (micritic) aggregates packed into burrows or accumulated at or near the surface opening of burrows can be interpreted as fecal products of invertebrate organisms^39^. At ODP Hole 696B, rounded- and lobate-shaped glauconitized pellets (Supplementary Fig. S4B,C), frequently filling cavities left by the burrowing activity (e.g. pellet-lined burrow *Ophiomorpha*; Supplementary Fig. S3C), were observed. Furthermore, pellets which can be interpreted as fecal in origin (Supplementary Fig. S3G) were observed underlying the glauconitic section (*Subunit VIID* of Barker *et al*.^17^: organic-rich, sandy mudstone facies including diverse calcareous faunas; see Supplementary Fig. S3F). Therefore, macroscopic and microscopic evidences suggest that glauconitized pellets deposited at the SOM shelf are likely of fecal origin. Glauconization is thought to be concomitant with rising sea levels, as it favours the successive availability of shallower substrates to be glauconitized^3^. In addition it allows for decreased terrigenous inputs preventing rapid burial that would inhibit the authigenic process. A similar evolutionary pattern is envisaged in the study area between the zone of production of pellets and the zone of major glauconitization. At Hole 696B, inner- to middle-shelf (20–100 m water depth) calcareous faunas (*Subunit VIID* of Barker *et al*.^17^; Supplementary Fig. S3F) underlie the glauconitic section, which attest to a very shallow marine environment prior major glauconitization. In addition, Owens and Sohl^42^ reported that Cretaceous-Tertiary low alumina glaucony from New Jersey-Maryland Coastal Plains characterizes the middle to outer shelf environment with depths of >50 m. The low Al concentrations of types 1 and 2 glaucony at Hole 696B (Al_2_O_3_ < 10%; Table 1) thus suggest water depths greater than 50 m. Furthermore, the abundance, maturity and evolution of the substrates subject to glauconitization fit well with most of the described features in condensed glauconitic sections formed at the shelf-slope transition^3^. Therefore, a continuous sea-level rise must have occurred in order to shift the glauconitization zone over the pelleted substrate deposited in the vicinity of the SOM shelf. Hence, we infer that the sea-level rise involved an upward reduction in the supply of clastic/siliciclastic detritus during glauconitization (e.g., glaucony maturation), ultimately leading to the formation of a transgressive condensed sediment sequence (Supplementary Fig. S7).

Microscopic features and geochemistry of glaucony grains provide additional insights about the environmental conditions prevailing during its formation. Environmental quiescence and low sedimentation rates favoured the appropriate physico-chemical regime to prevail for the Fe(III)-smectite-to-glauconite reaction (glaucony maturation) to occur at ODP Hole 696B (Fig. 7). Thus, sub-oxic and moderately reducing bottom water conditions near the sediment-water interface induced shallow burial diagenesis on a glaucony-bearing bioturbated substrate (Supplementary Figs S3C,D; S4A). Furthermore, winnowing must have been active at the SOM shelf during glauconitization to stir slightly the mud-fraction and provide sub-oxygenation allowing bioturbation. Sub-oxic conditions may have favoured the rapid degradation of organic matter along with the partial dissolution of detrital clay minerals, Fe-(oxy)hydroxides, K-feldspar, bio-opal and carbonates, leading to neoformation of the Fe(III)-smectite precursor for glauconite (Fig. 7). The singular occurrence of mineralized spheroidal and tubular capsules (i.e. bacterial remains on type 1 glaucony; Fig. 2F and Supplementary Fig. S4D) suggests that microbial activity played some role in the formation of the Fe(III)-smectite precursor. Similar processes are reported in a modern setting of the West Pacific Ocean^43^. Microbial activity has also been reported in glauconite-bearing sediments formed in different geological palaeogeographic settings, such as are the Jurassic-Cretaceous condensed sections of Tethyan margins^44,45^ or the epicontinental Upper Jurassic Georgiev Formation of the Western Siberia Basin^11,32,36^. It seems likely that microbial oxidation of organic matter was critical in creating favourable redox conditions for iron fixation in the octahedral layer of nascent glauconitic structures (Fig. 7).

The origin of the chemical elements necessary for glauconitization remains controversial. Normal seawater contains low concentrations of Fe, Al and Si, which do not provide favourable conditions for direct glauconite precipitation; in contrast, K concentrations are appreciable (~0.4 ppt^46^). Under these conditions, seawater is unlikely to contribute significant Fe to glauconitization but is a viable source of K. In addition, Fe in the present-day Southern Ocean is the primary factor limiting productivity, as determined by mesoscale Fe-enrichment experiments^47^. Therefore, continental supply related to physical weathering of terrigenous particles rich in Fe, K and Si is the most cited viable source for glauconitization in shallow marine sediments^31,48^. The Fe_2_O_3_ (total Fe) content of autochthonous types 1 and 2 glaucony (>27 wt%; Table 1) is higher than most reported open shelf glaucony-bearing deposits^13^. In modern oceans, high Fe-supply sources are likewise available in high-productivity outer shelf to slope upwelling zones, where glaucony often occurs in condensed horizons^49^. Several pathways can supply iron to the Fe-limited Southern Ocean at the present day^50^: estuarine and groundwater inputs, shelf/slope sediment resuspension, hydrothermalism, glacial runoff, sea-ice melt and atmospheric deposition. In the study area, we propose that the high Fe-enrichment of types 1 and 2 grains could be related to continent-derived Fe supply due to weathering of the adjacent South Orkney Islands (see location in Supplementary Fig. S2A). This enrichment can be either coetaneous to glauconitization at Hole 696B or be related to previous intense chemical weathering during Paleogene hyperthermals^21,51^. Alternatively, other processes could lead to the high Fe-enrichment of types 1 and 2 glaucony, such as high productivity induced by regional upwelling, or hydrothermal venting related to the Powell Ridge spreading axis^25,26^.

Glauconite cannot be directly precipitated at the K^+^ concentrations available in natural pore waters, but it can gradually evolve from Fe-rich smectite (either of detrital origin or of authigenic origin and precipitated in microbial settings^44,45^), through increased interlayer charge and K^+^ fixation due to loss of swelling layers and reduction of octahedral iron^44^. Localized acidification during early glauconitization (nascent stage; Fig. 7) promoted the formation of Fe(III) smectite. Acidification also favours halmyrolysis (submarine chemical alteration and/or dissolution at the sea floor), *e.g*. K leaching from the dissolution of biotite, muscovite and K-feldspar of the surrounding detrital sediment, which increases K content in pore waters. Consequently maturation of Fe-rich smectite to glauconite was triggered by K^+^ fixation under moderately reducing (post-oxic) conditions. The irreversible K^+^ fixation probably favoured the evolution from Fe(III)-smectite to glauconite in the same way as the illitization of smectite does^52^. The acidification related to the Fe(III)-smectite formation could be buffered by calcium carbonate dissolution, which is supported by replacement of foraminiferal tests by silica, as commonly observed in the studied core section (*Subunit VIIC*; *sensu* Barker *et al*.^17^). Experimental work has also shown that high silica concentrations are necessary for the Fe(III)-smectite precursor of glauconite to form, as Fe-rich, 7 Å clays (berthierine) will form instead at low silica concentrations^53^. The Southern Ocean, at present, plays a key role in the global production of biogenic silica^54^. The late Eocene witnessed also a biogenic silica radiation^55^, which resulted in high species diversity and enhanced Southern Ocean biogenic opal burial^56^. This may link glaucony formation in the SOM shelf to the presence of abundant primary bio-opal as revealed by HRTEM images (Fig. 3D).

The reported paleoenvironmental and paleoceanographic changes during and following the glauconitization process in the cores 57R-56R of the Hole 696B (ODP Leg 113) provide a window into changing conditions in the SOM from ~35.5 to 34.10 Ma, just before the EOT. Further work on late Eocene glaucony deposits from around the Antarctic margin will provide new insights into potential links between the Antarctic ice sheet development and the coeval changes in the tectonic and paleoceanographic configurations.

### Post-depositional alteration of glaucony at Hole 696B

The rare occurrence of type 3 glaucony, with greenish cores and altered brownish rims/cracks (Figs 2B, 5 and 6; Supplementary Figs S5D and S6) needs further explanation. The presence of zoned glauconitized grains has been explained either as a result of an alteration of glaucony (weathering)^52,57^, or as an intermediate stage of evolution from nascent to highly evolved glaucony (i.e. from glauconitic smectite to glauconitic mica^31^)^1,58^. At ODP Hole 696B, the typical textural form of glauconitic smectite found at glaucony rim-grains by Odom^58^ is not observed. In addition, the chemical alteration affecting type 3 glaucony is not restricted to the rims but it is also observed within the cores through cracks (e.g. Fig. 2D). EPMA point-by-point analyses of these grains indicate that K and Fe were leached from greenish mature (evolved glaucony) producing the Al-rich brownish rims and cracks (Supplementary Table S2). Brownish rims and cracks of type 3 glaucony grains are thus interpreted to form in relation to the development of a *secondary*, dioctahedral Fe(III)-smectite (nontronite) due to alteration after previous type 3 *primary* glaucony. This is supported by the *d*001 peak in XRD (Fig. 4A,B), mineral chemistry (Fig. 5; Supplementary Table S2 and Supplementary Fig. S6), and the HRTEM images (Figs 4C and 6). Pestitschek *et al*.^59^ suggested that weathering could reverse the glauconitization process, as glauconitic minerals degrade to smectite. The observed smectitization process leading to the formation of nontronite in the rims/cracks of type 3 glaucony could result thus from subaerial weathering in soils (reversion of the glauconitization process *sensu* Courbe *et al*.^57^; see also Eder *et al*.^11^) or as an oxidized product of the interaction with hydrothermal fluids (submarine weathering, or halmyrolysis^60^). There is no reported evidence of subaerial exposure of the SOM, but local raising along major syn-rift structures (*e.g*. graben/horst-bounding faults; Supplementary Fig. S2A) could trigger subaerial weathering on rift shoulders of raised/tilted blocks. Nontronite is common and widespread in modern hydrothermal vent areas^5,61^. Dredge samples from the southwestern margin of Powell Basin include middle Eocene hydrothermally-modified alkali basalts^26^ (Supplementary Fig. S2A), which suggest that the proto-Powell Basin could have started to open by continental extension in Eocene times. Rift-related volcanism in the proto-Powell Basin and major syn-rift structures at the SOM shelf, could have guided either subaerial weathering on tilted blocks or submarine hydrothermally triggered halmyrolysis, which would favour the alteration of previous formed glaucony grains.

Penecontemporaneous remobilization of parautochthonous (*sensu* Amorosi^38^) glaucony grains by waves, tides, storms or currents is common within shallow-marine to deep-water environments^62^. Type 3 glaucony thus most probably constitutes one type of intra-sequential grains that are altered (oxidized) from grains with similar characteristic to *in situ* type 2 glaucony. These altered grains may have been transported from nearby areas of the SOM shelf affected either by subaerial weathering or by hydrothermal vents and deposited next to *in situ* and non-altered grains of types 1 and 2. An alternative hypothesis could consider alteration of type 3 glaucony grains to have been synchronous with green-clay authigenesis at Hole 696B, but neither evidences of oxidation of autochthonous types 1 and 2 glaucony nor other mineral particles or matrix have been identified. Likewise, physical characteristics of type 3 grains evidence mechanical breakdown subject to transport from their place of formation.

### Further constraints: limiting factor for glauconitization

Subsequent transgression favoured burial, falling O_2_ levels, and ongoing reducing sulphidic conditions at Hole 696B during early diagenesis. The increasingly reducing conditions from suboxic-postoxic to sulphidic environments during deepening and sediment burial allowed enough production of S^2−^ in pore waters from marine SO_4_^2−^, which favoured the removal of Fe^2+^ for iron-bearing mineral precipitation. Diagenetic pyrite formed later, as revealed in petrographic and BSE images, either within the fine-grained matrix of the sediment (and often within the matrix surrounding the periphery of glaucony grains: e.g. Supplementary Fig. S5G) or infilling fissures within glaucony grains due to compaction (Supplementary Fig. S5H). Baldermann *et al*.^8^ stated that when Eh conditions within the fecal pellets micromilieu turn to low values and anoxic conditions, the green-clay forming process stops and pyrite forms. In our study site, iron-sulphide precipitation was thus a limiting factor for glauconitization by sequestration of Fe^2+^, as described in comparable glaucony-bearing facies^8,11,36^. A significant decrease in acidity of pore waters, as previously discussed, along with sediment porosity reduction and compaction with increased burial depth also favoured significant silica diagenesis at ODP Hole 696B (Fig. 3B–D). Consequently, sulphate reduction and silica diagenesis played a major role in diagenetic reactions observed throughout the glaucony-bearing facies in the studied section.

## Conclusions

The integrated sedimentological, mineralogical and geochemical characterization of glaucony facies at ODP Hole 696B assesses the physico-chemical conditions prevailing during glauconitization in the Antarctic late Eocene sediments. These conditions occurred in the open marine, shelf-slope transition of the SOM under sub-oxic moderately reducing conditions near to the sediment-water interface. The required environmental conditions were triggered by low sedimentation rates leading to stratigraphic condensation. Recurrent winnowing by bottom currents stirred the mud fraction slightly and provided sub-oxygenation at that site. This study represents the first well-documented case of late Eocene autochthonous, mature (evolved; K_2_O-rich ~7 wt%), smectite-poor interstratified ~10 Å glauconite-smectite occurrence in Antarctica. Therefore, glaucony authigenesis is utilized here as a reliable paleoenvironmental indicator for Antarctic Cenozoic climate history. Glaucony authigenesis thus marks the base of a transgressive condensed sequence deposited at the SOM shelf margin during the late Eocene. Results from this work provide important new insights into changing paleoceanographic conditions during a late Eocene transgressional event, just before the EOT. Further work is needed to understand the implications of this transgression for the investigation into the growth of the continent-wide ice sheet and/or the controversial opening/deepening of the Drake Passage.

## Supplementary information

Supplementary Information

## Data Availability

The datasets generated and/or analyzed during the current study are available from the corresponding author upon reasonable request.
