# Supplementary material for: Glaucony authigenesis, maturity and alteration in the Weddell Sea: An indicator of paleoenvironmental conditions before the onset of Antarctic glaciation
Source: Sci Rep. 2019 Sep 19;9:13580. doi: 10.1038/s41598-019-50107-1 (PMC6753099; doi:10.1038/s41598-019-50107-1)
Supplement: Supplementary file 1 — Supplementary Information [file 41598_2019_50107_MOESM1_ESM.pdf]

Glaucony authigenesis, maturity and alteration in the Weddell Sea: An indicator of paleoenvironmental conditions before the onset of Antarctic glaciation

Adrián López-Quirós[1*], Carlota Escutia[1], Antonio Sánchez-Navas[2], Fernando Nieto[2], Antonio García-Casco[2], Agustín Martín-Algarra[3], Dimitris Evangelinos[1], Ariadna Salabarnada[1]

[1]Instituto Andaluz de Ciencias de la Tierra, CSIC-Universidad de Granada, Avda. las Palmeras 4, 18100 Armilla, Granada, Spain; [2]Department of Mineralogy and Petrology, University of Granada, 18071 Granada, Spain; [3]Department of Stratigraphy and Paleontology, University of Granada, 18071 Granada, Spain. *(email: alquiros@iact.ugr-csic.es).

Supplementary information includes text and figures.

Supplementary text

Geological setting and lithostratigraphy

The South Orkney Microcontinent (SOM) is the largest (about 70000 km^2) continental fragment of the South Scotia Ridge between the Scotia and Antarctic plates, and is a remnant of the original link connecting the Antarctic Peninsula and South America (Fig. 1A). The SOM was disconnected from the Antarctic Peninsula along an E-W margin, probably during the Eocene and early Oligocene (40–30 Ma[1]). The SOM then continued to drift and rotate eastward until it reached its current position with respect to the Antarctic Peninsula during the early Miocene[1, 2]. The southeast and southwest SOM passive margins are connected to the proto-oceanic Powell and the oceanic Jane basins respectively, formed after the rifting between the Antarctic Peninsula and the SOM (Fig. 1B). The Airy, Bouguer and Eötvös basins structured in a sequence of horst and grabens (Supplementary Fig. S2A) during the Eocene/Oligocene extensional phase of the Scotia Ridge fragmentation[1]. In the northern part of the SOM, an east-west trend following the transform plate boundary, affected the structure of the basement high and the Newton Basin (Supplementary Fig. S2A). The South Orkney Islands are the only emerged area in the northern margin of the SOM shelf, comprising mainly Permian-Triassic sandstones and mudstones (metapelites and metagreywackes corresponding to the Greywacke Shale Formation/Scotia Metamorphic Complex) deposited by turbidity currents in a slope environment[3, 4].

Ocean Drilling Program (ODP) Hole 696B (61°50.96'S 42°56.00'W) was drilled at 650 m water depth on the southeastern margin of the SOM in the northwestern Weddell Sea (61°S; 42°W)[5] (see location in Supplementary Figs. S1 and S2A). At ODP Site 696, hemipelagic (0-214 mbsf), pelagic (214-530 mbsf), and terrigenous and authigenic (530 mbsf to the base of the hole) sediments were deposited between the late Eocene and the Quaternary[5, 6] (Supplementary Fig. S2B). This study focuses on two cores, 56R and 57R, recovered from the terrigenous section (shipboard *Unit VII*). Shipboard, the late Eocene-early Oligocene *Unit VII* (548 mbsf to base of hole at 646 mbsf) is divided in four subunits[5]. The dominant lithology of *Subunit VIID* (early late Eocene ~37.6-35.5 Ma; 645.6 to 606.9 mbsf) is characterized by organic-rich sandy mudstone facies. The sediments of *Subunit VIID* contain abundant macrofossil shells and shell fragments (e.g. Supplementary Fig. S3F). Late Eocene (~35.5-34.1 Ma) sediments of *Subunit VIIC* are characterized by high amounts of glaucony grains. This study focuses on the glaucony-bearing packstone facies of the lower part of this

lithological Subunit (cores 56R to 57R; Supplementary Fig. S2B-C), which appear condensed (see below). The upper part of this Subunit (latest Eocene; 569.7 to 579.4 mbsf) is characterized by packstone to wackestone facies. The major lithologies within *Subunit VIIB* (early Oligocene, ~34.1-33.2 Ma; 548.9 to 579.4 mbsf) are claystone and lime mudstone facies. All lithologies in *Subunit VIIB* contain minor amounts of glaucony grains.

Age assessments and sedimentation rates

Stratigraphic control of sediments in the lower part of the Hole 696B (*Subunit VIID)* was established through calcareous nannofossils (Supplementary Fig. S2B). The First Consistent Occurrence (FCO) of *Isthmolithus recurvus*[6] and the presence of *Reticulofenestra bisecta*[6] places the base of the terrigenous and authigenic sequence at ~36.5 Ma (643.62 mbsf) (*sensu* Villa *et al.*[7]), although the sequence could be as old as 37.6 Ma. The calcareous nannofossil *Reticulofenestra oamaruensis*[6] is recorded at the base of *Subunit VIIC* (588.72 to 598.42 mbsf) (Supplementary Fig. S2B), suggesting a maximum age of about 35.5 Ma (*sensu* Villa *et al.*[7]). For the overlying sediments, dinocysts provide an age of late Eocene to early Oligocene[8, 9] (Supplementary Fig. S2B). The First Occurrence (FO) of *Stoveracysta kakanuiensis* was calibrated to the latest Eocene (>33.8 Ma) in sediments/rocks from the East Tasman Plateau (ODP Site 1172) and in New Zealand[10]. In ODP Hole 696B, the FO of *S. kakanuiensis*[8, 9] (34.1 Ma; 571.55 mbsf) stratigraphically predates the FO of the Oi-1 marker *Malvinia escutiana*[8, 9] (569.11 mbsf), which suggests that the EOT was well recovered. The FO of the low-latitude taxon *Chiropteridium galea*[8] (552.2 mbsf) suggests a rather complete overlying lowermost Oligocene succession[11] (<33.26 Ma). Based on the biostratigraphic constraints, the studied glaucony-rich sediments (cores 56R and 57R; Supplementary Fig. S2B) are comprised between ~35.5 Ma and 34.1 Ma (i.e., predate the Oi-1 sediments) thus underlying the base of the early Oligocene.

The available biostratigraphic age model indicates a mean sedimentation rate of ~4cm/kyr in the lower part of the terrigenous Hole 696B, which covers the late Eocene (Supplementary Fig. S7). The mean sedimentation rate then decreases to ~1.85cm/kyr between 573 and 598 mbsf (encompassing most of the lithostratigraphic *Subunit VIIC;* Supplementary Fig. S7), which includes the latest Eocene glaucony-bearing interval. The well-recovered EOT appears to be a clear condensed section as sedimentation rates fell to an average of ~0.8cm/kyr. The overlying *Subunit VIIB,* spanning the early Oligocene, exhibits a mean sedimentation rate of about 4.41cm/kyr (Supplementary Fig. S7). These sedimentation rates, along with the depositional characteristics and lithologies, broadly agree with a condensed neritic sequence of poorly dated terrigenous glauconitic sediments as already suggested by Barker *et al.*[5]. Sequence condensation is highlighted by the age-depth plot, including not recovered intervals (average linear sedimentation rates indicated; Supplementary Fig. S7).

Supplementary Figures

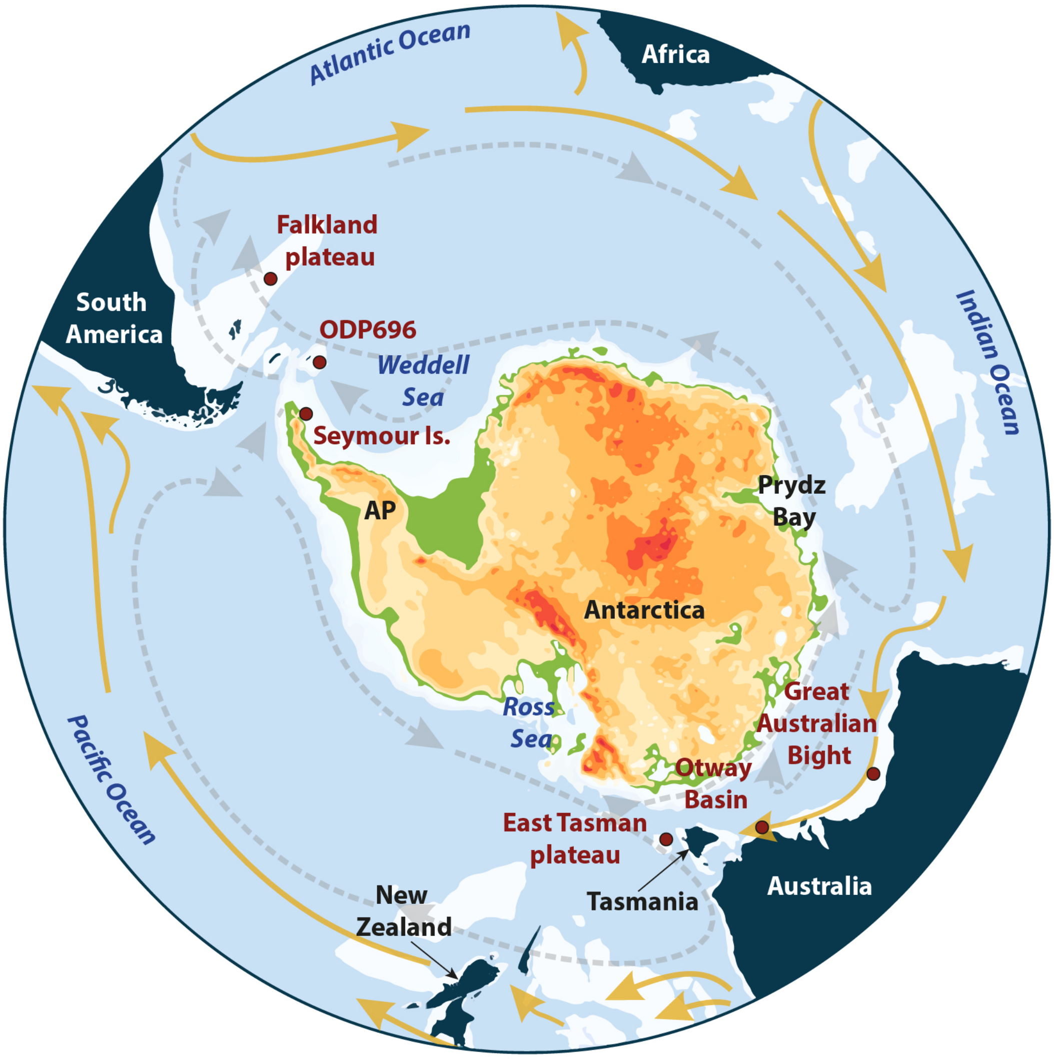

Supplementary Figure S1. Paleolocation of Eocene glaucony-bearing sections recorded around Antarctica. The Antarctic topography (Eocene; ~34 Ma) is from Wilson *et al.*[12]. White areas indicate submerged continental blocks and shelf environments, while green areas show lowland regions in Antarctica. Middle-late Eocene (~47-36 Ma) tectonic configuration is from Lawver and Gahagan[13]. Ocean circulation patterns (light grey arrows) are from Huber *et al.*[14], whereas low-latitude-derived currents (yellowish arrows) are from Houben *et al.*[8].

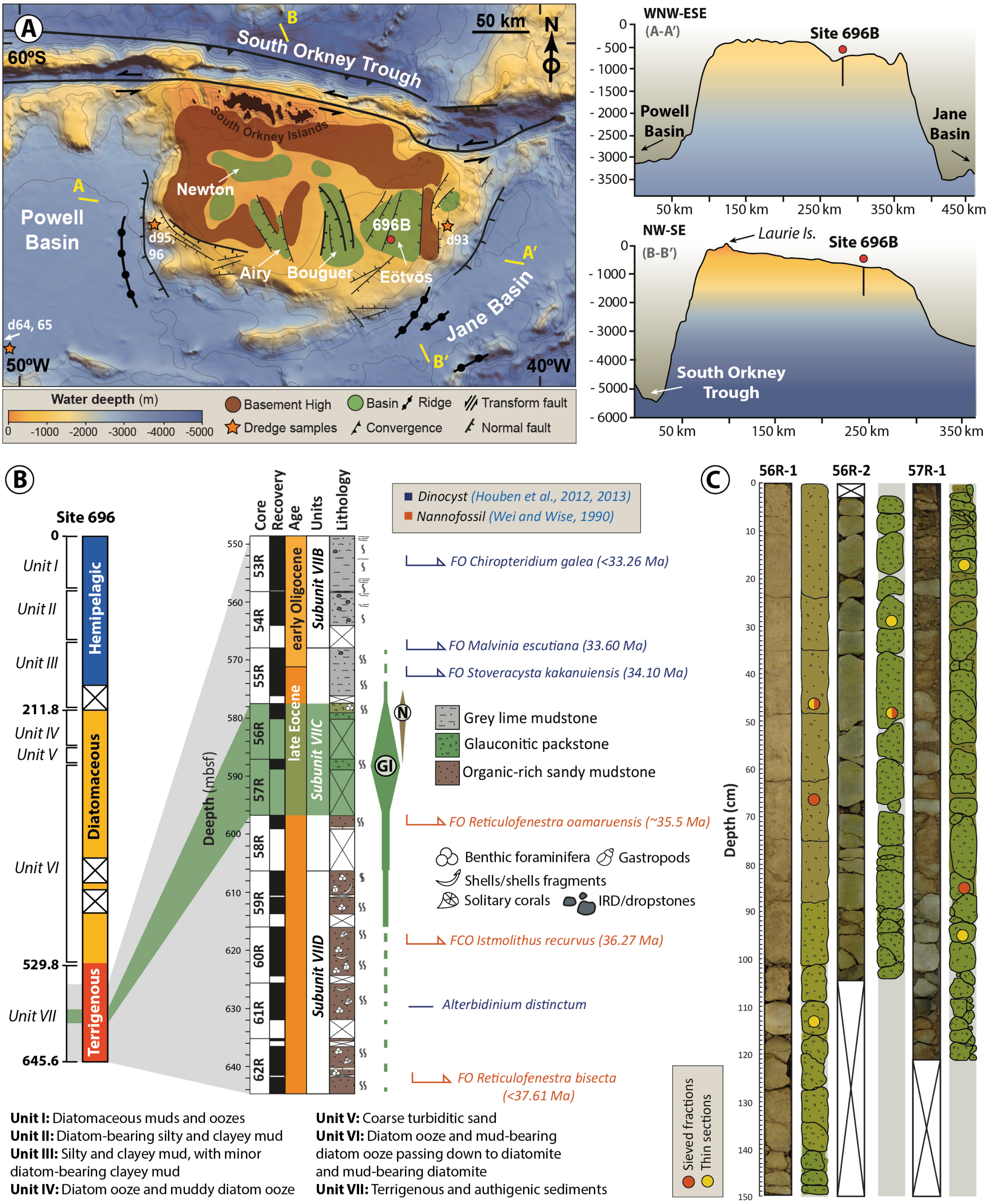

Supplementary Figure S2. A) Regional bathymetry of the South Orkney Microcontinent (SOM) shelf and the location of Hole 696B. Structural features displayed in the map extracted from King and Barker[1] and Busetti *et al.*[15]. Bathymetric map extracted from GEBCO 2014 (NOAA/NCEI). **B)** Simplified lithological log of the ODP Leg 113, Site 696 (from Barker *et al.*[5]). Enlarged area: detailed lithologic log of the *Subunit VII* (re-described after Barker *et al.*[5]). Main lithologies, facies and sedimentary structures are shown. Age constraints are from Barker *et al.*[5]; Wei and Wise[6]; Houben *et al.*[8, 9]). *Gl: glaucony; N: Nontronite after glaucony.* **C)** Line-scan digital images of the archive-half 56R and 57R investigated core sections. Red and yellow dots indicate the position of the samples studied.

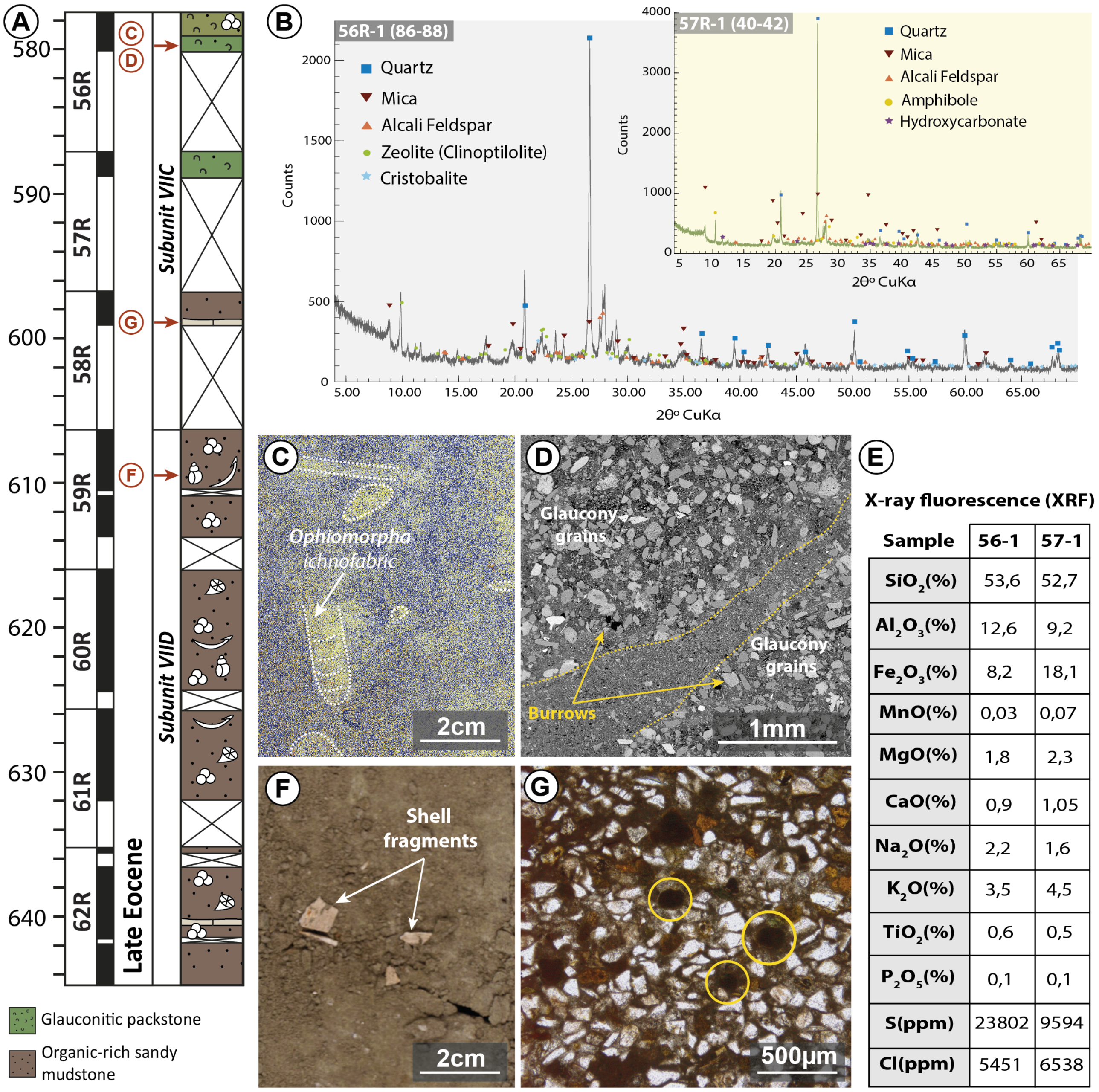

Sample	56-1	57-1
SiO_2(%)	53,6	52,7
Al_2O_3(%)	12,6	9,2
Fe_2O_3(%)	8,2	18,1
MnO(%)	0,03	0,07
MgO(%)	1,8	2,3
CaO(%)	0,9	1,05
Na_2O(%)	2,2	1,6
K_2O(%)	3,5	4,5
TiO_2(%)	0,6	0,5
P_2O_5(%)	0,1	0,1
S(ppm)	23802	9594
Cl(ppm)	5451	6538

Supplementary Figure S3. A) Detailed lithologic log displaying *subunits VIID* and *VIIC* (re-described after Barker *et al.*[5]). **B)** Representative X-ray diffraction patterns of bulk sediments from cores 56R-1 (86-88cm) and 57R-1 (40-42cm). **C)** Core slab of the glaucony-bearing facies (core 56R-1, 101-107cm) showing well-preserved burrows (e.g. pellet-lined burrow *Ophiomorpha*). Treated image from grayscale to the yellow-blue color channel to emphasise burrowing activity. **D)** BSE photomicrograph of burrowed glauconitic packstone facies. Note that glaucony accumulates at or near the surface opening of burrows. **E)** Representative bulk sediments composition by X-ray fluorescence (XRF) from cores 56R-1 (86-88cm) and 57R-1 (40-42cm). **F)** Core slab of characteristic organic-rich sandy mudstone facies (*Subunit VIID*) underlying the glauconitic section. **G)** Plane-polarized light (PPL) photomicrograph displaying the organic-rich, sandy mudstone facies. Note that yellow circles surround pellets which can be interpreted as faecal in origin.

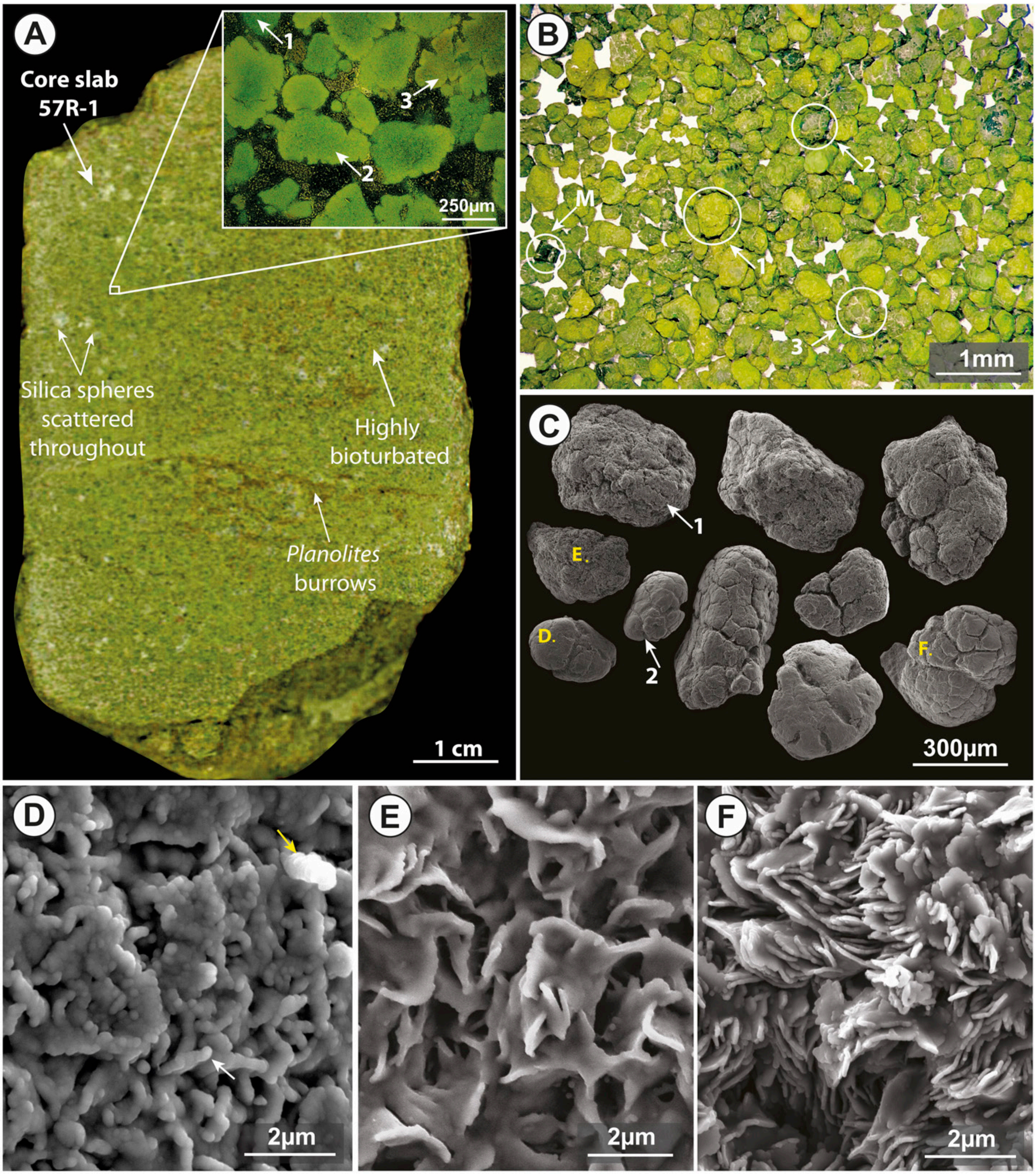

Supplementary Figure S4. **A)** Core slab of the glaucony-bearing facies (core 57R-1, 10-20cm) with burrowed (e.g., *Planolites*) light olive brown terrigenous matrix. Bioturbation and silica spheres (silicified foraminifers according to Barker *et al.*[5]) are observed throughout the cored glauconitic interval. Enlarged area: Reflected light microscope (RLM) photomicrograph displaying the different glaucony grain types. **B)** and **C)** Sieved and purified glaucony grains of types 1 and 2 (binocular glass and Secondary Electron (SE) photomicrographs, respectively). 1: type 1 glaucony, formed by irregular rounded smooth grains; 2: mammillated to lobate (cerebroid) type 2 glaucony; M: glauconitized mineral (mica)-grain. **D)**, **E)** and **F)** SE photomicrographs of enlarged areas in C, showing surface nanostructures of glaucony grains. D) Type 1 glaucony showing ill-defined globules (yellow arrow) and caterpillar (white arrow) structures resembling bacterial remains. E) Flaky nanostructures of type 1 glaucony. F) Flaky honeycombed and lamellar nanostructures of type 2 glaucony.

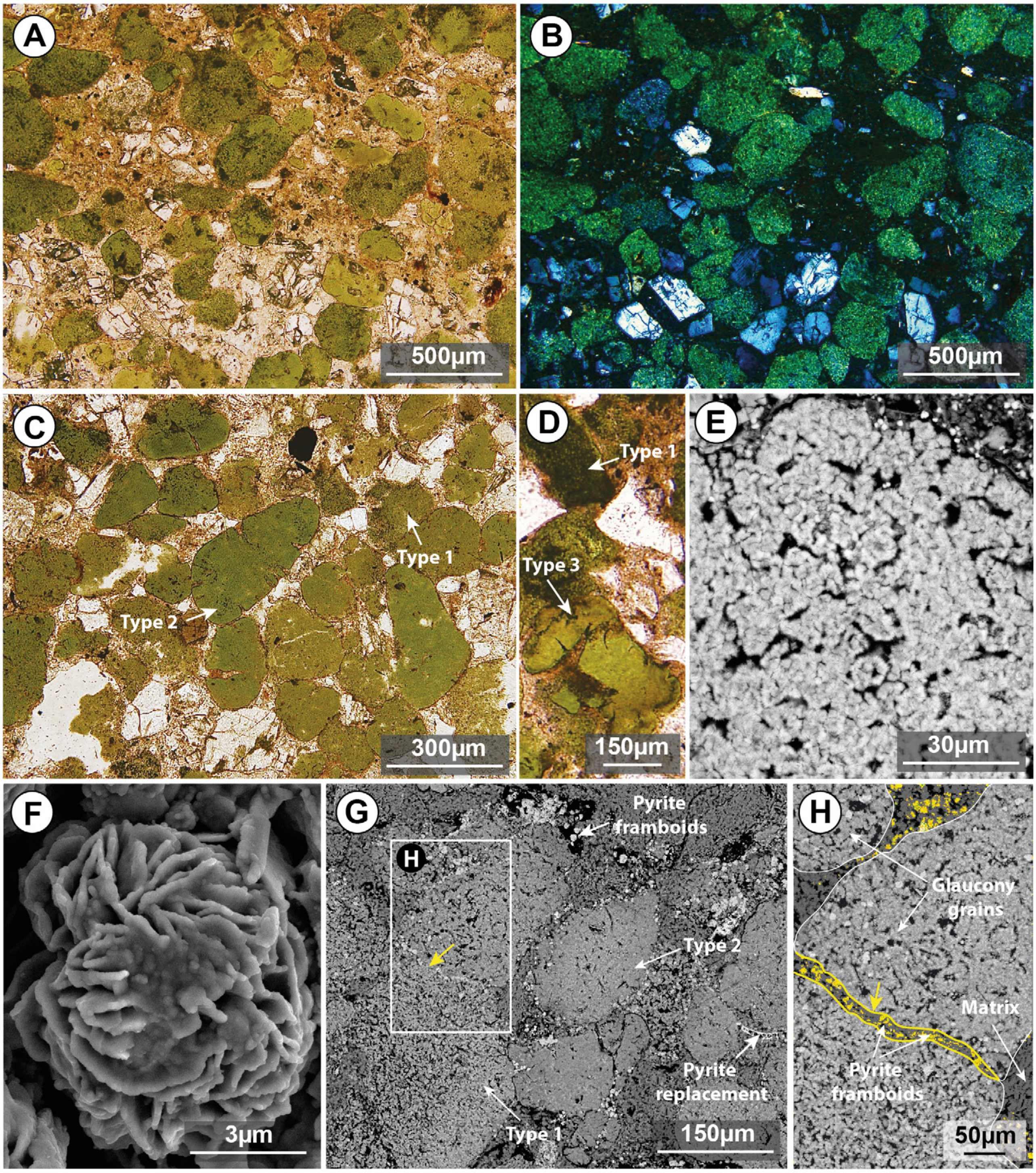

Supplementary Figure S5. A) and **B)** Plane-polarized light (PPL) and corresponding cross-polarized light (CPL) photomicrographs showing the studied glaucony packstone facies (*Subunit VIIC*). **C)** and **D)** PPL photomicrographs of glaucony types 1, 2 and 3. **E)** Back-scattered electron (BSE) photomicrograph of one slightly evolved to evolved type 1 glaucony grain. **F)** SE photomicrograph of the rosette nanostructure of one evolved type 2 glaucony grain. **G)** BSE photomicrograph of glaucony grains (displaying mainly flaky appearance of type 1 glaucony, and uniform compositions revealed by similar grey levels) surrounded by terrigenous matrix. Pyrite (i.e. framboids and pyrite replacements) observed throughout the terrigenous matrix. **H)** Enlarged area from G showing a crack partially filled with pyrite framboids (yellow arrow) also present within the matrix. Image treated to emphasise pyrite occurrence (yellow spots; false color) through the BSE compositions revealed by grey levels.

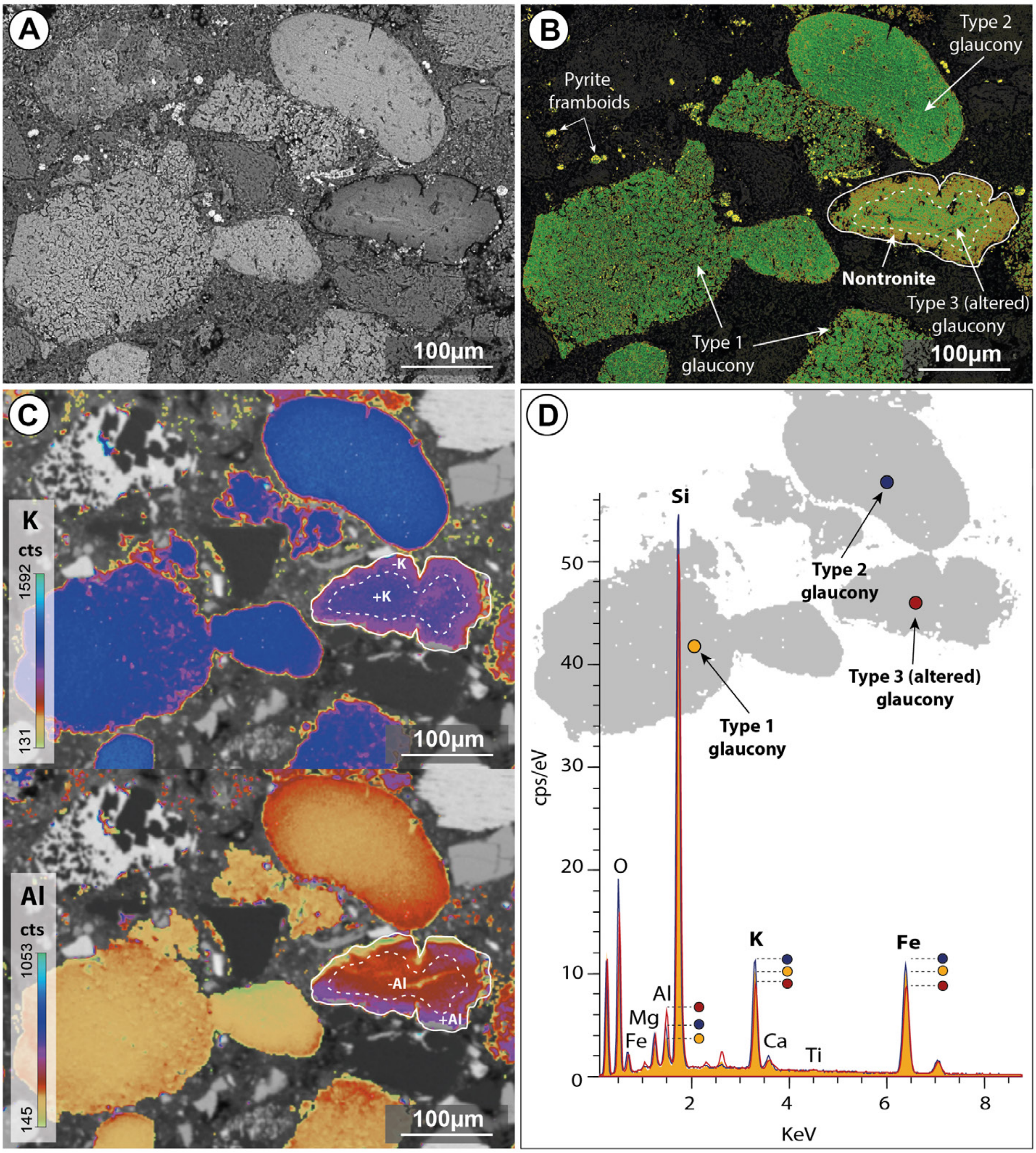

Supplementary Figure S6. A) BSE photomicrograph and **B)** corresponding treated image to emphasise pyrite (yellow) and glaucony (green to brown) occurrences from the black matrix (through the BSE compositions revealed by grey levels in A). Types 1, 2 and 3 glaucony are distinguished by subtle differences in grey (A) and in green to brown levels (B), which indicate slight differences in composition of each textural element or part of it. **C)** X-ray (Kα) maps of the same area shown in A (obtained by EPMA), showing abundances of K and Al with voids and polish defects, and with all other mineral phases masked out. Colors are superimposed onto a grey-scale base-layer calculated from the expression ∑ [(counts)i·Ai], (where A is atomic number, and i is the intensity of the X-Ray signal for Si, Ti, Al, Fe, Mn, Mg, Ca, Ba, Na, K, P, S and O), which contains the basic textural information of the scanned areas. Color scales represent counts (cts), with coldest colors indicating more intense X-ray signals (concentration). Rims of type 3 glaucony (nontronite) display lower K, and higher Al content relative to its cores and to types 1 and 2 glaucony. **D)** Energy-dispersive X-ray (EDX) analysis of types 1, 2 and 3 glaucony. The yellow, blue and red dots indicate the position of the EDX analysis in the glaucony grains shown in A, B and C.

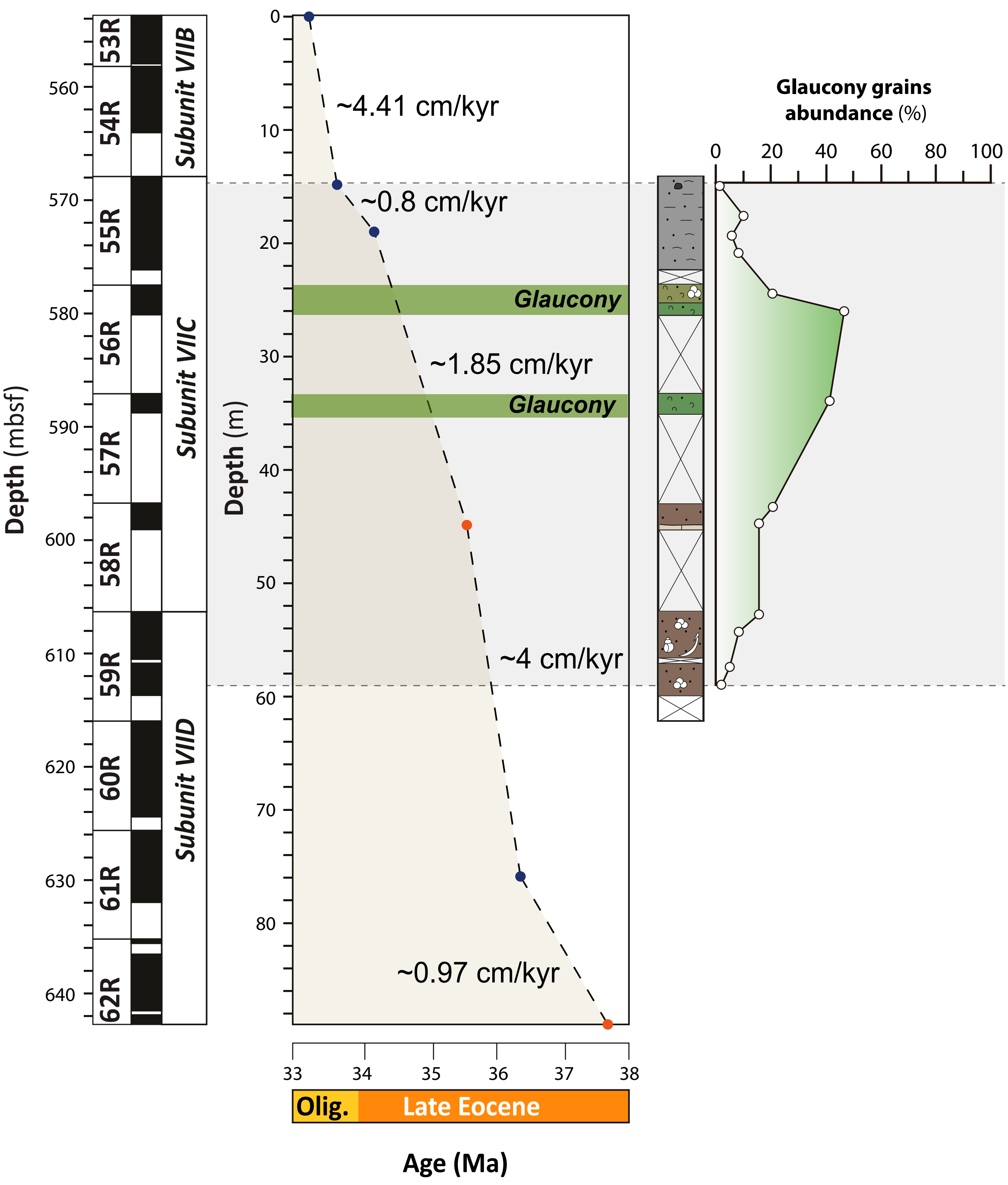

Supplementary Figure S7. Estimated sedimentation rate for the terrigenous to authigenic sediments of *Subunit VII* (Hole 696B). Average linear sedimentation rates indicated. Core sections and biostratigraphic markers: calcareous nannofossils (orange dots) and dinocysts (blue dots) are indicated. Abundance of glaucony grains (%) is also indicated.

Supplementary Table S1. Electron-microprobe (EPMA) analyses and [1]calculated formulae obtained for types 1 and 2 glaucony grains from thin section. [1]Units: atoms p.f.u. Normalization to 2 octahedral + 4 tetrahedral cations and 22 charges (10 O+2 OH). *Total Fe expressed as Fe^{3+}; † Fe^{2+} and Fe^{3+} in the formula calculated by stoichiometry.

**Type**	1	1	1	1	1	1	1	1	1	1	2	2	2	2	2	2	2	2	Mica	Mica
Chemical composition in w%																				
SiO_2	51.82	52.23	51.50	47.73	51.05	51.90	51.64	53.76	52.63	48.67	51.53	49.28	52.87	51.23	50.15	53.14	52.53	51.16	49.43	47.62
TiO_2	0.21	0.18	0.20	0.22	0.18	0.24	0.28	0.23	0.15	0.17	0.16	0.50	0.15	0.48	0.18	0.10	0.15	0.17	0.25	0.28
Al_2O_3	2.57	2.62	2.33	3.12	3.08	2.92	3.37	3.45	2.95	2.73	3.47	4.68	2.75	3.89	2.99	3.22	3.06	3.02	3.95	3.03
*Fe_2O_3	28.08	27.60	28.49	24.31	26.64	28.21	26.87	26.45	27.35	26.15	26.80	26.59	27.61	26.33	29.69	27.61	27.16	27.79	26.72	26.27
MnO	0.00	0.01	0.00	0.00	0.00	0.00	0.00	0.01	0.00	0.00	0.00	0.00	0.01	0.00	0.01	0.00	0.00	0.00	0.00	0.00
MgO	3.03	3.08	3.24	2.93	2.94	3.11	3.24	3.10	3.31	2.80	3.40	2.91	3.30	2.86	2.98	3.11	3.05	2.96	3.25	2.91
CaO	0.26	0.25	0.33	0.09	0.32	0.19	0.31	0.38	0.31	0.05	0.25	0.14	0.24	0.23	0.26	0.25	0.24	0.26	0.16	0.13
Na_2O	0.05	0.08	0.10	0.09	0.09	0.08	0.08	0.06	0.05	0.10	0.08	0.08	0.06	0.09	0.08	0.09	0.10	0.12	0.06	0.08
K_2O	7.34	7.36	6.95	6.46	6.41	7.55	7.06	6.67	7.17	6.62	7.51	7.82	7.20	6.96	7.86	7.36	6.98	6.58	7.62	7.07
H_2O	4.03	4.04	4.03	3.71	3.96	4.07	4.04	4.12	4.08	3.79	4.05	3.97	4.09	4.01	4.03	4.12	4.06	4.01	3.96	3.77
Total	97.40	97.45	97.17	88.66	94.66	98.26	96.89	98.23	98.01	91.08	97.25	95.99	98.29	96.07	98.23	98.99	97.31	96.05	95.40	91.16
Atoms per formula unit (a.p.f.u.) on the basis of 22 charges																				
Si	3.85	3.88	3.83	3.86	3.87	3.83	3.83	3.91	3.86	3.85	3.82	3.72	3.87	3.83	3.73	3.87	3.88	3.83	3.74	3.78
^{IV}Al	0.15	0.12	0.17	0.14	0.13	0.17	0.17	0.09	0.14	0.15	0.18	0.28	0.13	0.17	0.26	0.13	0.12	0.17	0.26	0.22
Σ^{IV}	4	4	4	4	4	4	4	4	4	4	4	4	4	4	4	4	4	4	4	4
Ti	0.01	0.01	0.01	0.01	0.01	0.01	0.02	0.01	0.01	0.01	0.01	0.03	0.01	0.03	0.01	0.01	0.01	0.01	0.01	0.02
^{VI}Al	0.08	0.11	0.04	0.15	0.14	0.08	0.13	0.2	0.12	0.1	0.12	0.13	0.11	0.17	0	0.14	0.15	0.1	0.1	0.07
†Fe^{3+}	1.3	1.25	1.39	1.26	1.29	1.31	1.28	1.18	1.27	1.33	1.28	1.3	1.29	1.23	1.45	1.24	1.25	1.37	1.36	1.36
†Fe^{2+}	0.28	0.29	0.21	0.21	0.23	0.25	0.22	0.27	0.24	0.22	0.21	0.21	0.24	0.25	0.21	0.27	0.26	0.2	0.16	0.21
Mn	0	0	0	0	0	0	0	0	0	0	0	0	0	0	0	0	0	0	0	0
Mg	0.34	0.34	0.36	0.35	0.33	0.34	0.36	0.34	0.36	0.33	0.38	0.33	0.36	0.32	0.33	0.34	0.34	0.33	0.37	0.35
Σ^{VI}	2	2	2	2	2	2	2	2	2	2	2	2	2	2	2	2	2	2	2	2
Ca	0.02	0.02	0.03	0.01	0.03	0.02	0.02	0.03	0.02	0	0.02	0.01	0.02	0.02	0.02	0.02	0.02	0.02	0.01	0.01
Na	0.01	0.01	0.01	0.01	0.01	0.01	0.01	0.01	0.01	0.02	0.01	0.01	0.01	0.01	0.01	0.01	0.01	0.02	0.01	0.01
K	0.7	0.7	0.66	0.67	0.62	0.71	0.67	0.62	0.67	0.67	0.71	0.75	0.67	0.66	0.75	0.68	0.66	0.63	0.74	0.72
Σ^{XII}	0.72	0.73	0.7	0.69	0.66	0.74	0.71	0.66	0.7	0.69	0.74	0.78	0.7	0.7	0.78	0.72	0.69	0.67	0.76	0.74

Supplementary Table S2. Results of electron-microprobe (EPMA) analyses and [1]calculated formulae obtained for altered type 3 glaucony (rim- and core-grains) from thin sections. [1]Units: atoms p.f.u. Normalization to 2 octahedral + 4 tetrahedral cations and 22 charges (10 O+2 OH). *Total Fe expressed as Fe^{3+}; † Fe^{2+} and Fe^{3+} in the formula calculated by stoichiometry.

**Type**	3-rim	3-rim	3-rim	3-rim	3-rim	3-rim	3-rim	3-core	3-core	3-core	3-core	3-core	3-core	3-core	3-core	3-core
Chemical composition in wt%																
SiO_2	47.16	46.78	48.79	47.08	53.77	49.26	48.71	51.15	50.88	51.65	54.01	54.75	51.98	54.06	52.26	50.53
TiO_2	0.24	0.30	0.33	0.33	0.28	0.29	0.34	0.32	0.31	0.30	0.26	0.23	0.21	0.25	0.20	0.16
Al_2O_3	5.55	4.70	4.08	5.02	6.88	5.98	4.42	5.86	4.81	5.38	5.63	5.98	3.72	4.62	3.55	2.95
$^*Fe_2O_3$	17.85	19.71	23.43	22.32	19.86	19.08	21.65	21.40	23.84	21.44	22.32	21.96	25.22	24.21	25.65	26.57
MnO	0.00	0.00	0.01	0.00	0.00	0.00	0.00	0.00	0.00	0.00	0.00	0.01	0.00	0.00	0.01	0.00
MgO	2.88	3.26	3.13	3.15	3.34	3.22	3.38	3.52	3.10	3.58	3.48	3.45	3.12	3.21	3.07	3.03
CaO	0.21	0.34	0.19	0.24	0.58	0.30	0.49	0.40	0.36	0.26	0.42	0.46	0.40	0.39	0.33	0.38
Na_2O	0.19	0.17	0.17	0.13	0.22	0.15	0.10	0.07	0.09	0.15	0.08	0.11	0.06	0.08	0.11	0.09
K_2O	4.33	4.86	5.84	5.53	4.81	4.84	5.25	5.24	5.82	5.34	5.40	5.45	6.14	6.30	6.39	6.13
H_2O	3.58	3.61	3.81	3.73	4.10	3.78	3.77	3.98	3.96	3.98	4.14	4.18	4.01	4.13	4.02	3.93
Total	81.99	83.73	89.78	87.53	93.82	86.89	88.11	91.93	93.16	92.10	95.74	96.56	94.85	97.26	95.59	93.77
Atoms per formula unit (a.p.f.u.) on the basis of 22 charges																
Si	3.95	3.89	3.84	3.78	3.93	3.91	3.87	3.85	3.85	3.89	3.91	3.93	3.89	3.92	3.9	3.86
^{IV}Al	0.05	0.11	0.16	0.22	0.07	0.09	0.13	0.15	0.15	0.11	0.09	0.07	0.11	0.08	0.1	0.14
Σ^{IV}	4	4	4	4	4	4	4	4	4	4	4	4	4	4	4	4
Ti	0.02	0.02	0.02	0.02	0.02	0.02	0.02	0.02	0.02	0.02	0.01	0.01	0.01	0.01	0.01	0.01
^{VI}Al	0.5	0.35	0.22	0.25	0.53	0.46	0.28	0.37	0.28	0.37	0.39	0.43	0.22	0.32	0.21	0.12
$†Fe^{3+}$	0.99	1.13	1.25	1.3	0.94	1.03	1.17	1.16	1.21	1.13	1.09	1.03	1.21	1.08	1.2	1.33
$†Fe^{2+}$	0.14	0.1	0.14	0.05	0.16	0.1	0.12	0.05	0.15	0.08	0.13	0.15	0.21	0.25	0.24	0.19
Mn	0	0	0	0	0	0	0	0	0	0	0	0	0	0	0	0
Mg	0.36	0.4	0.37	0.38	0.36	0.38	0.4	0.4	0.35	0.4	0.38	0.37	0.35	0.35	0.34	0.35
Σ^{VI}	2	2	2	2	2	2	2	2	2	2	2	2	2	2	2	2
Ca	0.02	0.03	0.02	0.02	0.05	0.03	0.04	0.03	0.03	0.02	0.03	0.04	0.03	0.03	0.03	0.03
Na	0.03	0.03	0.03	0.02	0.03	0.02	0.01	0.01	0.01	0.02	0.01	0.02	0.01	0.01	0.02	0.01
K	0.46	0.52	0.59	0.57	0.45	0.49	0.53	0.5	0.56	0.51	0.5	0.5	0.59	0.58	0.61	0.6
Σ^{XII}	0.51	0.57	0.63	0.61	0.53	0.54	0.59	0.55	0.6	0.56	0.54	0.55	0.63	0.63	0.65	0.64

Supplementary references

1. King, E. C. & Barker, P. F. The margins of the South Orkney microcontinent. *Journal of the Geological Society* **145**, 317–331 (1988).

2. Coren, F. *et al.* Morphology, seismic structure and tectonic development of the Powell Basin, Antarctica. *Journal of the Geological Society London* **154**, 849–862 (1997).

3. Dalziel, I.W.D. Tectonic evolution of a fore-arc terrane, southern Scotia Ridge, Antarctica. *Geological society of America Special Publications* **200**, 1–32 (1984).

4. Flowerdew, M. J., Riley, T. R. & Haselwimmer, C. E. Geological Map of the South Orkney Islands (1:1 50 000 scale). BAS GEOMAP 2 Series, Sheet 3 (British Antarctic Survey, 2011).

5. Barker, P. F. *et al*. Proceedings of the Ocean Drilling Program, *Initial Reports*, 113. Ocean Drilling Program, College Station, Texas (1988).

6. Wei, W. & Wise, S. W. Middle Eocene to Pleistocene calcareous nannofossils re- covered by Ocean Drilling Program Leg 113 in the Weddell Sea. In: Proceedings of the Ocean Drilling Program, *Scientific Results* 113, vol. 188. Ocean Drilling Program, College Station, Texas, 639–666 (1990).

7. Villa, G., Fioroni, C., Pea, L., Bohaty, S. & Persico, D. Middle Eocene-late Oligocene climate variability: Calcareous nannofossil response at Kerguelen Plateau, Site 748. *Marine Micropaleontology* **69**, 171–192 (2008).

8. Houben, A. J. P. Triggers and consequences of glacial expansion across the Eocene-Oligocene Transition. Utrecht, the Netherlands: LPP Contribution Series No. 39 (Utrecht University, 2012).

9. Houben, A. J. P. *et al*. Reorganization of Southern Ocean plankton ecosystem at the onset of Antarctic Glaciation. *Science* **340**, 341–344 (2013).

10. Clowes, C. D. Stoveracysta, a new gonyaulacacean dinoflagellate genus from the upper Eocene and lower Oligocene of New Zealand. *Palynology* **9**, 27–35 (1985).

11. Pross, J. *et al*. Umbria – Marche revisited: A refined magnetostratigraphic calibration of dinoflagellate cyst events for the Oligocene of the Western Tethys. *Review of Palaeobotany and Palynology* **158**, 213–235 (2010).

12. Wilson, D. S. *et al*. Antarctic Topography at the Eocene-Oligocene Boundary. *Palaeogeography, Palaeoclimatology, Palaeoecology* **335–336**, 24–34 (2011).

13. Lawver, L. A. & Gahagan, L. C. Evolution of Cenozoic seaways in the circum-Antarctic region. *Palaeogeography, Palaeoclimatology, Palaeoecology* **198**, 11–37 (2003).

14. Huber, M. *et al*. Eocene circulation of the Southern Ocean: Was Antarctica kept warm by Subtropical waters? *Paleoceanography* **19**, PA4026, doi:10.1029/2004PA001014 (2004).

15. Busetti, M., Marchetti, A., Zanolla, X., De Cillia, C. & Belyaev, V. Tectonic history of the south Orkney Microcontinent from seismic structure and stratigraphy. *Royal Society of New Zealand Bulletin* **35**, 507–513 (2002).
